# Using Electronic Clinical Quality Measure Reporting for Public Health Surveillance

**Published:** 2015-05-01

**Authors:** Dawn Heisey-Grove, Hilary K. Wall, Amy Helwig, Janet S. Wright

**Affiliations:** 1 Office of Planning, Evaluation, and Analysis, Office of the National Coordinator for Health Information Technology; 2 Division for Heart Disease and Stroke Prevention, National Center for Chronic Disease Prevention and Health Promotion, CDC; 3 Agency for Healthcare Research and Quality

By June 2013, three fourths of office-based practicing physicians in the United States had adopted some form of electronic health record (EHR) system (1). With greater EHR use, more health data are linked with available patient demographic information in a format that is easily retrievable and collected at the point of care (2). This highlights the potential of electronic clinical quality measure (CQM) reporting data for use in monitoring population health for those receiving health care services. To assess this possibility, electronic CQM data that were submitted to the Medicare EHR Incentive Program were analyzed to assess provider progress toward achieving blood pressure control among their patients with hypertension. Approximately 63,000 health care providers reported at least 1 time over 3 years, representing approximately 17 million patients with hypertension. On average, 62% of patients with hypertension had controlled blood pressure. Use of EHR data for public health surveillance could streamline reporting, facilitating more timely and possibly more complete data collection in key areas of public health concern.

Recent adoption of EHR systems was encouraged by monetary incentives for participation and financial penalties for noncompliance under the Health Information Technology for Economic and Clinical Health (HITECH) Act of 2009 (3,4). Through the HITECH Act, the Medicare EHR Incentive Program provided financial incentives for the adoption and meaningful use of certified EHR technology (5). To receive a meaningful use incentive payment, eligible health care providers must attest that they used their certified EHR system in a way that improved patient safety, care coordination, quality and efficiency of care, and public health reporting, as well as in a way that encouraged patient engagement and protected the privacy and security of sensitive health information (6).

The first attestations were reported to the Centers for Medicare & Medicaid Services in 2011. Those attestations were required to include a report of performance on six CQMs, most of which were endorsed by the National Quality Forum (NQF). The aggregate numerators and denominators for these CQMs were required to be calculated by a certified EHR system of the health care provider. CQMs were required so that providers would become familiar with generating population-level quality data from their EHR systems. For this reason, CQMs could be developed in the EHR system by either the vendor or health care practice staff without additional validation, no minimum performance thresholds were established for these measures, and providers could report a value of zero for either the numerator or denominator without penalty. CQMs were reported in aggregate to protect patient privacy and reflect the population of patients seen by the eligible health care provider and for whom data were entered into the EHR. Because these data reflect all patients seen by the provider during a given measure’s reporting period, they represent a useful resource for population-level surveillance for key public health concerns for persons receiving care. To avoid missing an incentive payment, eligible providers were required to attest each year after their initial attestation; to avoid penalties in 2015, all Medicare-eligible providers were required to demonstrate meaningful use by 2014.

Providers demonstrating meaningful use had to report on three required CQMs and three additional CQMs selected from a list of optional measures. Several of those optional CQMs were aligned with clinical performance goals of Million Hearts, a U.S. Department of Health and Human Services initiative that was launched in 2012 to prevent 1 million heart attacks and strokes by 2017. One strategy of Million Hearts is to help clinicians and health care systems focus on achieving excellence in a small set of evidence-based CQMs.* To reach the 2017 goal, Million Hearts set a clinical performance target of ≥70% for each of these CQMs. This is first example of electronic CQM data being used to evaluate nationwide progress toward a public health improvement goal.

The number of health care providers reporting Million Hearts CQMs through the incentive program has steadily increased over time, whereas the percentage of Medicare attestations for which the optional measures were chosen has remained relatively constant (Table 1). The highest proportion of eligible health care providers reported the blood pressure control measure (National Quality Forum [NQF] #0018†; 26%–27%), compared with the taking aspirin when appropriate measure (NQF #0068; 3%–4%) and the cholesterol management measure (NQF #0064; 17%–19%). Therefore, only NQF #0018 is discussed in this report.

Approximately 63,000 health care providers reported at least 1 time over 3 years on their progress toward achieving blood pressure control among their patients with hypertension (NQF #0018), representing approximately 17 million patients with hypertension. On average, 62% of patients with hypertension had controlled blood pressure; this percentage remained stable during 2011–2013 at 62%–63% (Table 2). At least one third of all Medicare attesters reporting on this measure controlled the blood pressure of ≥70% their patients with hypertension. Differences were found between early and later adopters in the incentive program (Table 3). Differences across the various attestation cohorts varied slightly; however, the differences were not statistically significant in 2013, when all three groups reported. The early adopters (2011 cohort) had a large proportion of health care providers who were at or above the Million Hearts clinical performance goal, and performance in the 2011 cohort did not change significantly during the 3 years of reporting. The 2012 cohort’s performance improved slightly (p<0.001) from their first to their second attestation.

## Discussion

The data in this report represent a large sample of both physicians and patients and not only allow for ascertainment of aggregate annual performance on measures of public health concern but also for longitudinal studies based on a large patient population. The performance reported for these measures is similar to the performance measured through the Healthcare Effectiveness Data and Information Set (HEDIS), which reflects blood pressure control (NQF #0018) performance on the basis of health insurance plan reporting (7). HEDIS 2012 values ranged from 57% to 69% across the different plans. The values reported through the incentive program CQMs fall within that range. The incentive program CQM reporting includes all patients seen by the provider. As a result, the population and overall performance might be expected to differ from the HEDIS data slightly because the incentive program CQMs might include patients not covered by health insurance or who had inconsistent coverage over the course of a calendar year. Improvements in blood pressure control result from specific actions by clinicians and patients. Efforts to enhance medication adherence, support healthy habits, and provide training for self-monitoring will engage and enable patients to safely achieve and maintain control.

This report has at least seven limitations. First, NQF #0018 relies on the use of *International Classification of Diseases, Ninth Revision, Clinical Modification* code 401, Essential Hypertension, to generate the measure denominator. Therefore, patients who exhibited clinical characteristics of hypertension but did not have an official hypertension diagnosis were not counted, possibly resulting in an overestimate of true blood pressure control in the patient population. In contrast, the National Health and Nutrition Examination Survey, considered the gold standard for estimating national rates of blood pressure control among the entire U.S. population, estimated blood pressure control at 52% during 2011–2012 (8). Second, participating health care providers might not be representative of non-Medicare, or nonparticipant, provider populations. Third, because providers were able to select from various quality measures, they might have reported on measures for which they had better performance, although no financial incentive to do so was available. Fourth, because these data were reported in aggregate by individual health care providers, certain patients might have been counted twice if they were cared for by multiple providers. Fifth, CQM data were self-reported and might not have been validated by an Office of the National Coordinator for Health Information Technology Authorized Certification Body. To increase data validity, starting in 2014, incentive program CQM reporting must be performed using an EHR that has been certified to calculate that measure (9). Sixth, incentive program CQM reporting was based only on the data available in the EHR system of the health care provider. If a patient transitioned to another provider, such as a specialist, the original EHR might not have subsequent, possibly improved, blood pressure values recorded. Increased electronic exchange of health information, in which patient health information is reported back to the primary care provider, might ensure that the primary care provider is aware of such improvements in the patient’s health. Finally, CQM data do not include patient-reported blood pressure values. Therefore, if a health care provider was monitoring blood pressure for certain patients using patient-reported data, those patient-reported data were not included Understanding how to use patient-reported data for decision-making is critical as more patients engage in self-monitoring.

What is already known on this topic?

New data sources can be used by public health organizations to streamline population health surveillance, increase the timeliness of data collection, and decrease associated expenses. Health care providers participating in the Centers for Medicare & Medicaid Services Medicare Electronic Health Record (EHR) Incentive Program are reporting electronic clinical quality measures (CQMs) for all patients. These electronic CQMs can be used to monitor various population health issues.

What is added by this report?

Electronic CQMs reported through the Medicare EHR Incentive Program were used to measure whether eligible health care providers met clinical performance goals set by the Million Hearts initiative, an initiative established to prevent 1 million heart attacks and strokes by 2017. Approximately 63,000 health care providers reported at least 1 time during 2011–2013, representing approximately 17 million patients with hypertension. On average, 62% of patients with hypertension had controlled blood pressure. One third of health care providers met the Million Hearts clinical performance goal of controlling the blood pressure ≥70% of their patients with hypertension.

What are the implications for public health practice?

Population health surveillance can be costly, time consuming, and limited, depending on the data source. CQMs reported to the Medicare EHR Incentive Program reflect aggregate data on all patients seen by a health care provider during a given measure’s reporting period and therefore represent a substantial proportion of the U.S. population. These data are reported as a function of another federal program and are the result of automated extraction from an EHR, which might streamline the reporting process for the health care provider, resulting in data that are a useful resource in public health surveillance.

Incentive program CQMs are calculated by extracting structured data elements collected in the EHR at the point of care, a process that reduces the amount of data retrieval required for tracking progress. In addition, alignment of CQMs across federal and private sector programs enables clinicians to collect data once and report to selected programs. This analysis demonstrates the potential for electronic CQM reporting to be used for monitoring population health. State and local public health agencies can partner with state, regional, or local health information exchanges; the state primary care association; the state Medicaid program; and health systems to explore the use of existing EHR data for surveillance while still ensuring appropriate safeguards to maintain patient privacy. Federal public health and health care agencies can collaborate to improve the strength and usability of EHR data as appropriate infrastructure at the state and local levels is being built and interoperability standards are being developed. As EHR implementation becomes more widespread, the data collected by these systems will be invaluable for monitoring numerous clinical conditions.

## Figures and Tables

**TABLE 1:** Number of providers reporting clinical quality measures through the Medicare Electronic Health Record Incentive Program and percentage of attestations for each CQM, by reporting year — United States, 2011–2013

Million Hearts goal	Corresponding CQM	CQM definition	Year	Reporting Medicare providers (no.)	Medicare attestations (%)
Aspirin when appropriate	NQF #0068	Percentage of patients who were discharged alive in past year with AMI, CABG, or PTCA or who had a diagnosis of IVD during measurement year who had documentation of aspirin or other antithrombotic during measurement year	2011	2,067	4
			2012	5,539	3
			2013	8,350	4
Blood pressure control	NQF #0018	Percentage of patients aged 18–85 years with a hypertension diagnosis who had adequate blood pressure control during measurement year	2011	14,968	26
			2012	48,644	26
			2013	62,928	27
Cholesterol management	NQF #0064	Percentage of patients aged 18–75 years with diabetes (type 1 or type 2) who had LDL-C <100 mg/dL	2011	11,094	19
			2012	33,577	18
			2013	40,807	17

**Abbreviations:** AMI = acute myocardial infarction; CABG = coronary artery bypass graft; CQM = clinical quality measure; IVD = ischemic vascular disease; LDL-C = low-density lipoprotein cholesterol; NQF = National Quality Forum; PTCA = percutaneous transluminal coronary angioplasty.

**TABLE 2:** Number of patients with hypertension, percentage of providers who reached the Million Hearts goal,* and percentage of patients with adequately controlled blood pressure,† by reporting year — United States, 2011–2013

Measure§	2011	2012	2013
**No. of patients with hypertension (millions)**	2	8	17
**Providers who reached Million Hearts Goal (%)**	41	36	36
**Patients with adequately controlled blood pressure (%)**			
Mean	63	61	62
First quartile	52	51	53
Median	66	64	65
Third quartile	77	75	75

**Abbreviations:** CQM = clinical quality measure; NQF = National Quality Forum.

* Controlled the blood pressure of ≥70% of patients with hypertension.

† <140/90 mmHg.

§ Among providers reporting CQM NQF #0018, which is defined as the percentage of patients aged 18–85 years who had a diagnosis of hypertension within the first 6 months of the measurement period, or any period of time before the measurement period, whose blood pressure was adequately controlled (<140/90 mmHg) during the measurement period.

**TABLE 3:** Percentage of patients with adequately controlled blood pressure,* by attestation cohort year†— United States, 2011–2013

	2011			2012			2013		
			
Cohort	No. of providers attesting§	Mean score (%)	% at Million Hearts goal¶	No. of providers attesting§	Mean score (%)	% at Million Hearts goal¶	No. of providers attesting§	Mean score (%)	% at Million Hearts goal¶
2011 cohort (early adopters) (N = 57,677)	14,968	63	41	13,811	62	36	14,353	63	39
2012 cohort (N = 138,848)	—	—	—	34,833	61	36	32,769	63	36
2013 cohort (later adopters) (N = 69,141)	—	—	—	—	—	—	15,806	60	33

**Abbreviations:** CQM = clinical quality measure; NQF = National Quality Forum.

* Among providers reporting CQM NQF #0018, which is defined as the percentage of patients aged 18–85 years who had a diagnosis of hypertension within the first 6 months of the measurement period, or any period of time before the measurement period, whose blood pressure was adequately controlled (<140/90 mmHg) during the measurement period.

† Eligible health care providers were assigned to an attestation cohort based on the first year they attested to meaningful use with the Medicare Incentive Program.

§ Across any 2 years in the program, 16%–18% attrition has occurred; however, most health care providers return to participate in subsequent years. In addition, because NQF #0018 is an optional CQM, health care providers may choose to report on the measure in 1 year and not in another.

¶ Controlled the blood pressure of ≥70% of patients with hypertension.

## Footnotes

* Available at http://millionhearts.hhs.gov/aboutmh/cqm_measure_alignment.html.

† Defined as the percentage of patients aged 18–85 years who had a diagnosis of hypertension within the first 6 months of the measurement period, or any period of time before the measurement period, whose blood pressure was adequately controlled (<140/90 mmHg) during the measurement period.
